# Consistency of spatial dynamics of HIV-1 and HCV among HIV-1/HCV coinfected drug users in China

**DOI:** 10.1186/s12879-021-06711-6

**Published:** 2021-09-25

**Authors:** Yu Wang, Xin Chen, Mei Ye, Wei Pang, Chiyu Zhang, Si-Dong Xiong, Yong-Tang Zheng

**Affiliations:** 1 Key Laboratory of Animal Models and Human Disease Mechanisms of the Chinese Academy of Sciences, Kunming Institute of Zoology, Chinese Academy of Sciences, 32 Jiaochang Donglu, Kunming, 650223 China; 2 KIZ-SU Joint Laboratory of Animal Models and Drug Development, College of Pharmaceutical Sciences, Soochow University, 199 Renai Road, Wuzhong District, Suzhou, 215000 China; 3 Department of Pathogenic Biology, School of Basic Medical Sciences, Gannan Medical University, Ganzhou, China; 4 Kunming College of Life Science, University of Chinese Academy of Sciences, Kunming, Yunnan China; 5 Shanghai Public Health Clinical Center, Fudan University, Shanghai, China; 6 Jiangsu Key Laboratory of Infection and Immunity, Institutes of Biology and Medical Sciences, Soochow University, Suzhou, China

**Keywords:** HIV-1, HCV, Spatial dynamic, Coinfection, Drug users, Yunnan

## Abstract

**Background:**

As the transmission routes of human immunodeficiency virus type 1 (HIV-1) and hepatitis C virus (HCV) are similar, previous studies based on separate research on HIV-1 and HCV assumed a similar transmission pattern. However, few studies have focused on the possible correlation of the spatial dynamics of HIV-1 and HCV among HIV-1/HCV coinfected patients.

**Methods:**

A total of 310 HIV-1/HCV coinfected drug users were recruited in Yingjiang and Kaiyuan prefectures, Yunnan Province, China. HIV-1 *env*, *p17*, *pol* and HCV *C/E2*, *NS5B* fragments were amplified and sequenced from serum samples. The genetic characteristics and spatial dynamics of HIV-1 and HCV were explored by phylogenetic, bootscanning, and phylogeographic analyses.

**Results:**

Among HIV-1/HCV coinfected drug users, eight HCV subtypes (1a, 1b, 3a, 3b, 6a, 6n, 6v, and 6u) and two HIV-1 subtypes (subtype B and subtype C), three HIV-1 circulating recombinant forms (CRF01_AE, CRF07_BC and CRF08_BC), and four unique recombinant forms (URF_BC, URF_01B, URF_01C and URF_01BC) were identified. HCV subtype 3b was the most predominant subtype in both Yingjiang and Kaiyuan prefectures. The dominant circulating HIV-1 subtypes for drug users among the two areas were CRF08_BC and URF_BC. Maximum clade credibility trees revealed that both HIV-1 and HCV were transmitted from Yingjiang to Kaiyuan.

**Conclusions:**

The spatial dynamics of HIV-1 and HCV among HIV-1/HCV coinfected drug users seem to have high consistency, providing theoretical evidence for the prevention of HIV-1 and HCV simultaneously.

**Supplementary Information:**

The online version contains supplementary material available at 10.1186/s12879-021-06711-6.

## Background

At the end of 2019, the total numbers of people living with human immunodeficiency virus type 1 (HIV-1) and hepatitis C virus (HCV) worldwide were estimated to be 38 and 71 million, respectively (http://www.who.int). As both HIV-1 and HCV can transmit through blood, sexual contact and mother to child, HIV-1/HCV coinfection is common worldwide. A global systematic analysis showed that in HIV-infected individuals, HIV-1/HCV coinfection was 82.4% in people who injected drugs (PWIDs) [1]. In China, the prevalence of HIV-1/HCV coinfection among PWIDs varied from 3.4% to 21.4% [2]. Previous studies have found that the prevalence of HIV-1/HCV coinfection is 15% among PWIDs in Yunnan province, and a high proportion can be seen in Yingjiang and Kaiyuan prefectures [3].

In China, current circulating HIV-1 subtypes comprise subtype B, subtype C, circulating recombinant form (CRF) 01_AE, CRF07_BC, CRF08_BC and other CRFs and unique recombinant forms (URFs). HIV-1 CRF01_AE is mainly transmitted through sexual contact and originates from Thailand, while HIV-1 subtypes C and B are mainly transmitted through injection of drugs and originate from India and Thailand, respectively. The three subtypes all spread to Yunnan province and further to other provinces [4, 5]. HIV-1 CRF07_BC and CRF08_BC were first identified among drug users in Yunnan province [6], and the two HIV-1 recombinants spread rapidly among drug users in China. The most prevalent HCV isolates in China are genotypes 1, 2, 3 and 6 [7–9]. Different HCV subtypes had diverse transmission routes, such as subtypes 3b, 6n, and 6u originating from Yunnan and Guangxi and subtypes 2a and 6a originating from southern China (e.g., Guangdong), which further spread to Jiangsu [10]. Zhang and colleagues found that HCV 3a spread from Xinjiang to Jiangsu and from Yunnan to Guangxi; these routes were similar to the routes of HIV-1 CRF07_BC and CRF08_BC [11]. The study indicated that there may be a correlation between HIV-1 and HCV, but it did not focus on the same population. A previous study identified the origin and transmission patterns of HIV-1 and HCV in former blood donor (FBD) patients with coinfections. The results showed that HIV-1 infections in FBDs were introduced from Thailand and HCV infections were introduced from Japan [12]; however, this study did not analyze the correlation between HIV-1 and HCV. Here, our study documented that eight HCV subtypes and nine HIV-1 subtypes were found among HIV-1/HCV coinfected drug users and that there was a possible association in transmission patterns between HIV-1 and HCV.

## Methods

### Study population

**Fig. 1:**
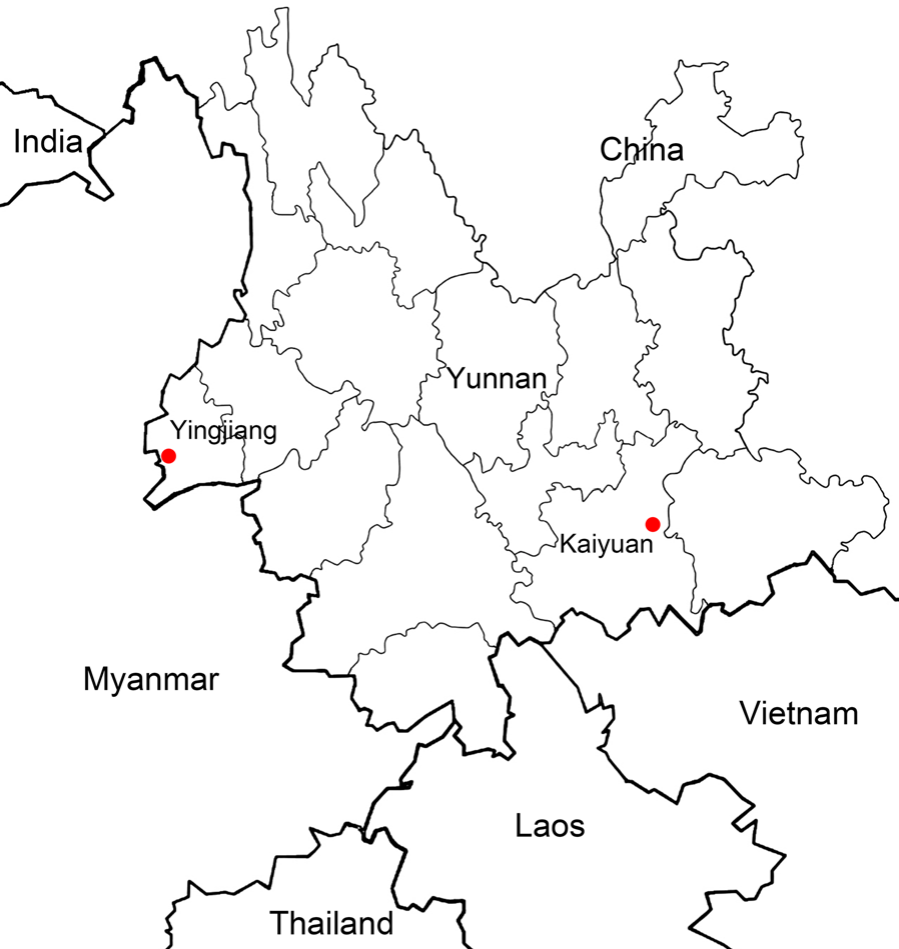
The geographical location of Yingjiang and Kaiyuan Prefectures, Yunnan Province, China

A cross-sectional study was conducted among drug users recruited from communities and methadone maintenance treatment programs with the assistance of local Centers for Disease Control and Prevention (CDC) in the Yingjiang and Kaiyuan prefectures of Yunnan province between 2009 and 2011 (Fig. 1). The study recruited 310 HIV-1/HCV coinfected drug users (183 from Kaiyuan prefecture and 127 from Yingjiang prefecture). Information regarding demographic characteristics, such as age, gender, and ethnicity, was collected from the interview. Venous blood was collected, centrifuged, and stored in a − 80 °C freezer.

### Amplification of HIV-1 and HCV gene fragments

Viral RNA was extracted from the plasma of HIV-1/HCV coinfected drug users using a High Pure Viral RNA Kit (Roche, Mannheim, Germany) and then subjected to amplification of HIV-1 *env*, *p17*, *pol* and HCV *C/E2* and *NS5B* genomic fragments. Reverse transcription PCRs were performed using the PrimeScript™ II 1st Strand cDNA Synthesis Kit (TaKaRa Biotechnology, Dalian, China), and then the PCR products were subjected to nested PCR using TransTaq DNA Polymerase High Fidelity (Beijing TransGen Biotech Co., Ltd., Beijing, China). The primer pairs used in the nested PCR were modified from previous papers [11–13]. The nested PCR products were sequenced with an ABI PRISM 377XL DNA sequencer (Applied BioSystems, California, America).

### Phylogenetic analyses of HIV-1 and HCV sequences

The HIV-1 and HCV sequences were aligned with a set of reference sequences available at Los Alamos National Laboratory HIV-1 Database and HCV Database using ClustalW. The alignment was edited using BioEdit 5.0.9. The gaps were removed manually, and the sequences were trimmed to obtain fragments of equivalent length. Phylogenetic trees were generated using the maximum-likelihood method implemented in MEGA 7.0 software, and the branch significance was analyzed by bootstrapping with 1000 replicates. To determine HIV-1 recombination, bootscan analyses were performed using SimPlot 3.5.1 software.

### Phylogeographic analysis of HIV-1 and HCV sequences

To investigate the possible correlation of HIV-1 and HCV among HIV-1/HCV coinfected drug users, reference HIV-1 *p17* and HCV *NS5B* sequences from prefectures outside Yingjiang and Kaiyuan in Yunnan were downloaded from the HIV-1 and HCV databases. Subsequently, HIV-1 subtype C (including the sequences of HIV-1 CRF07_BC and CRF08_BC, as their *p17* fragments were subtype C) and HCV 3b sequences were selected according to the results of the maximum-likelihood tree analysis and the online tool “Recombinant Identification Program” available in HIV-1 database. Bayesian phylogeographic analysis was then performed using BEAST 1.6.2 as previously described [11, 14, 15]. The accession numbers of reference sequences downloaded from GenBank and used for Bayesian phylogeographic analysis are listed in Additional file 1: Table S1 and Additional file 2: Table S2.

### Sequence data

The sequences reported in this article are available in GenBank under accession numbers MF990907-MF991024. MG334013-MG334120, MG385895-MG386024, MG432013-MG432106, MG450396-MG450541, MG461702-MG461853, MG494384-MG494479, MG549076-MG549188, MG549193-MG549305, MG763455-MG763560.

## Results

### Demographic characteristics

Most of the 183 HIV-1/HCV coinfected samples from Kaiyuan prefecture were from men who were of Han ethnicity, unemployed and single and who had an education level of secondary school (Table 1). Most of the 127 HIV-1/HCV coinfected samples from Yingjiang prefecture were from men who were of Dai ethnicity, farmers and single and who had an education level of primary school (Table 1).

**Table 1:** The demographic information of HIV-1/HCV coinfected drug users in Yingjiang and Kaiyuan prefectures, Yunnan, China

Variables	Yingjiang No. (%)	Kaiyuan No. (%)
Age		
Mean (95%CI^#^)	34.0 (18.5, 49.1)	37.9 (26.0, 49.8)
Gender		
Male	126 (99.2)	140 (76.5)
Female	1 (0.8)	43 (23.5)
Ethnicity		
Han	46 (36.2)	146 (79.8)
Hui	0	18 (9.8)
Dai	54 (42.5)	0
Jingpo	23 (18.1)	0
Yi	1 (0.8)	14 (7.7)
Others*	3 (2.4)	5 (2.7)
Occupation		
Farmer	102 (80.3)	0
Employed	1 (0.8)	13 (7.1)
Unemployed	24 (18.9)	170 (92.9)
Marriage status		
Single	57 (44.9)	90 (49.2)
Married/live with partner	46 (36.2)	65 (35.5)
Divorced	24 (18.9)	28 (15.3)
Education level		
None	18 (14.2)	4 (2.2)
Primary school	59 (46.5)	50 (27.3)
Secondary school	42 (33.1)	107 (58.5)
High school/university	8 (6.3)	22 (12.0)

^#^95% confidence interval; *Achang, Bai, Hani, Li, Lisu, Zhuang

Of the participants in Kaiyuan, 42 were self-reported as undergoing antiretroviral therapy (ART), 62 were ART-naïve, and 79 had not answered the questions regarding the status of ART; the numbers in Yingjiang were 26, 33, and 68, respectively. Information on patients receiving anti-HCV therapy was not collected in the present study.

### Subtype characterization of HIV-1 and HCV strains

**Fig. 2:**
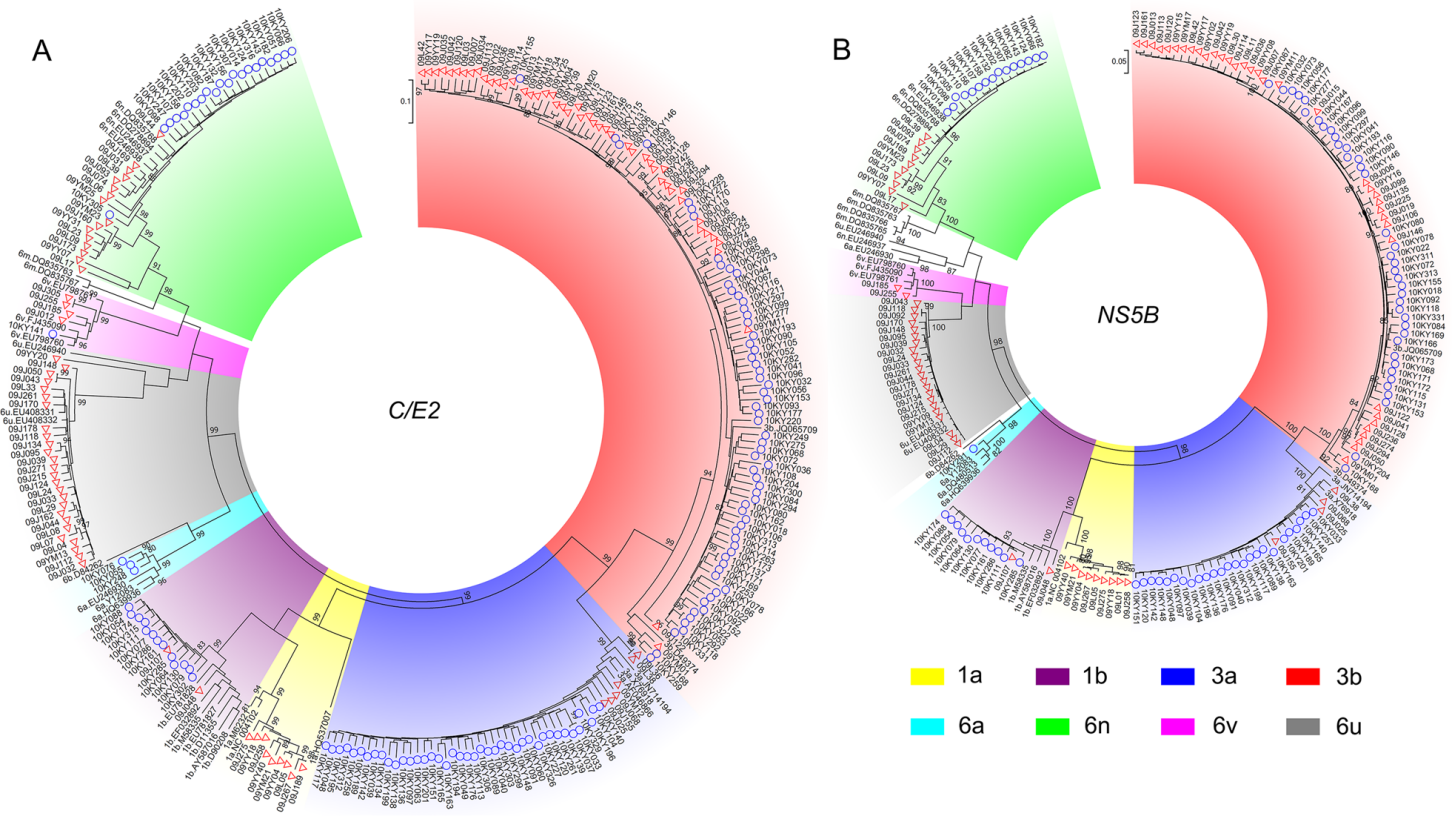
The maximum-likelihood trees based on *C/E2* (**A**) and *NS5B* (**B**) fragments of HCV among HIV-1/HCV coinfected drug users in Yunnan Province, China. The red triangles and the blue circles indicate the sequences that amplified from drug users in Yingjiang and Kaiyuan Prefectures, respectively. The different colored sectors indicate the sequences with different subtypes

**Fig. 3:**
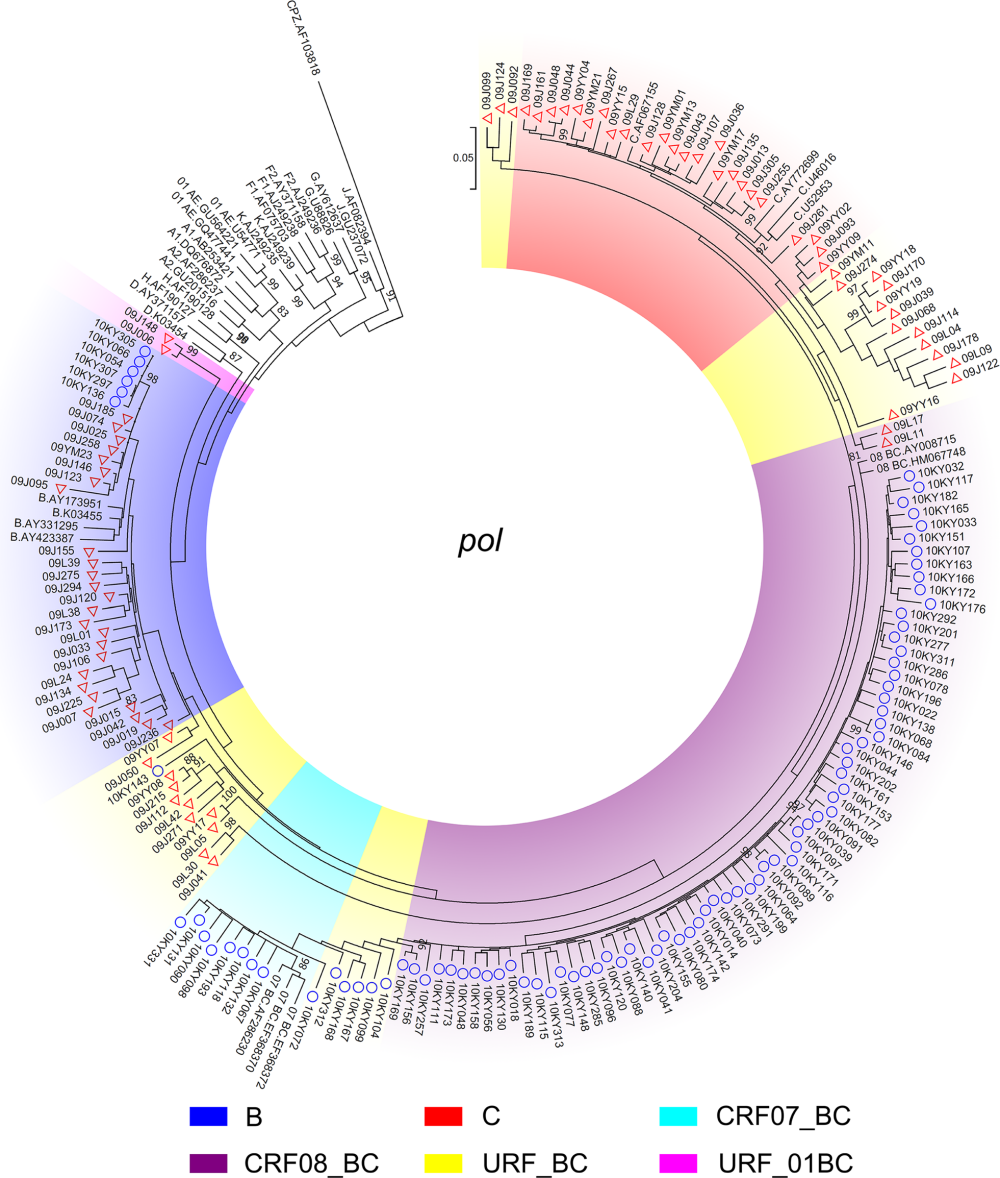
The maximum-likelihood trees based on *pol* fragments of HIV-1 among HIV-1/HCV coinfected drug users in Yunnan Province, China. The red triangles and the blue circles indicate the sequences that amplified from drug users in Yingjiang and Kaiyuan Prefectures, respectively. The different colored sectors indicate the sequences with different subtypes

Among the 183 HIV-1/HCV coinfected samples from Kaiyuan prefecture, 84.2% and 70.0% of them were successfully amplified with at least one fragment of the HCV *C/E2*, *NS5B* and HIV-1 *p17*, *pol*, *env* genes, respectively (Additional file 3: Table S3). Maximum-likelihood trees showed that HCV 3b was predominantly based on HCV C/E2 and NS5B, followed by 3a, 6n, 1b, 6a and 6v (Fig. 2). HIV-1 subtype C was predominantly based on HIV-1 *p17* and *env* fragments, followed by subtype B and CRF01_AE (Additional file 4: Figure S1). While for the HIV-1 *pol* fragments, CRF08_BC was predominant (Fig. 3). Taken together, as the *p17* fragments of CRF07_BC and CRF08_BC is subtype C, the results showed that HCV 3b (45.4%) and HIV-1 CRF08_BC (66.1%) were the most predominant subtypes among HIV-1/HCV coinfected drug users from Kaiyuan prefecture.

Among 127 HIV-1/HCV coinfected samples from Yingjiang prefecture, 90.6% and 89.8% of them were successfully amplified with at least one fragment of the HCV *C/E2*, *NS5B* and HIV-1 *p17*, *pol*, *env* genes, respectively (Additional file 3: Table S3). Maximum-likelihood trees showed that HCV 3b was predominantly based on HCV *C/E2* and *NS5B*, followed by 6u, 6n, 1a, 3a, 6v and 1b (Fig. 2). HIV-1 subtype C was predominantly based on HIV-1 *p17* and *env* fragments, followed by subtype B and CRF01_AE (Additional file 1: Figure S1). While for the HIV-1 *pol* fragments, URFs comprising HIV-1 subtypes B and C (URF_BC) were predominant (Fig. 3). Taken together, as the *p17* fragments of CRF07_BC and CRF08_BC is subtype C, the results showed that HCV 3b (43.4%) and URF_BC (53%) were the most predominant subtypes among HIV-1/HCV coinfected drug users from Yingjiang prefecture.

### Spatial dynamics of HIV-1 and HCV

**Fig. 4:**
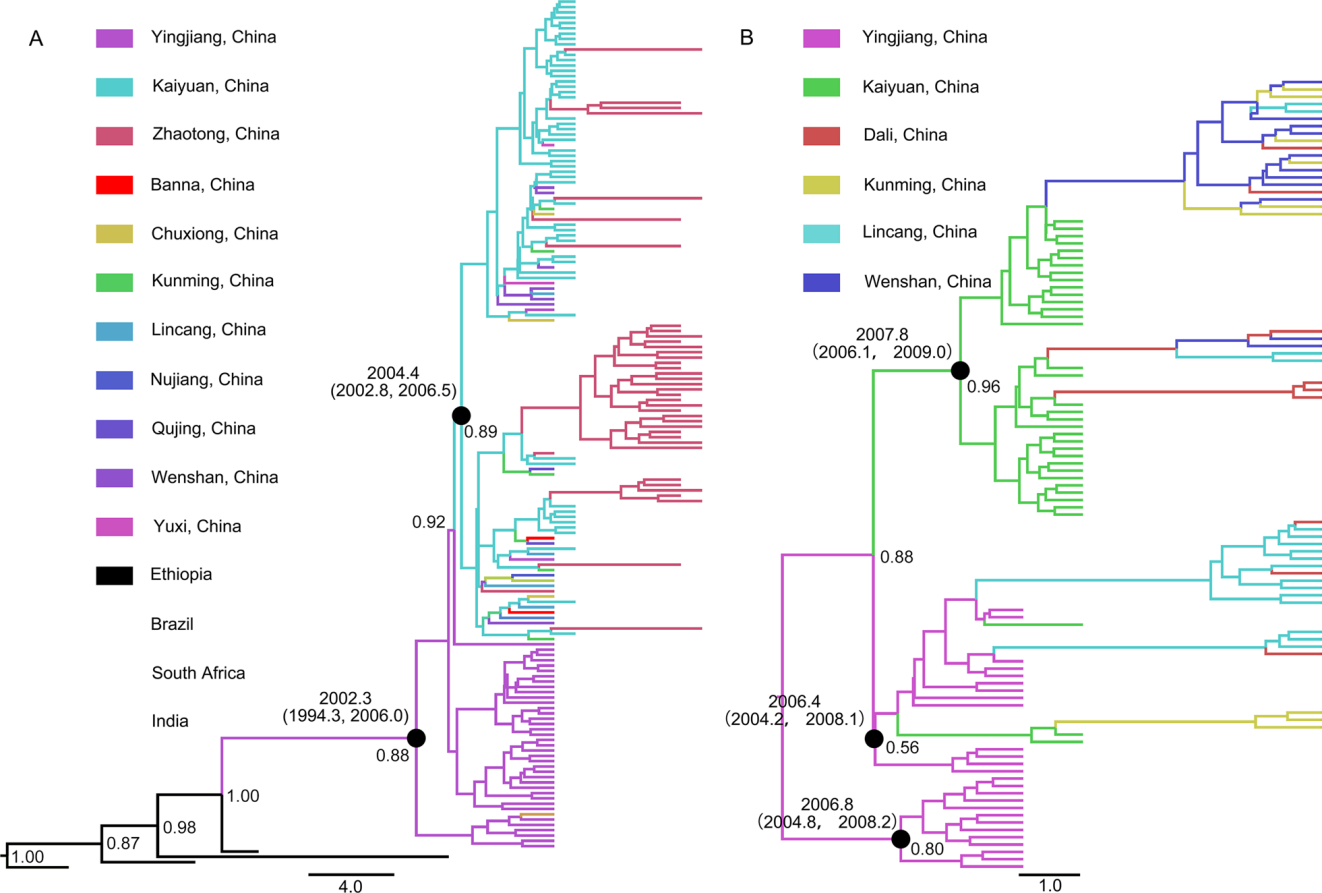
The Maximum clade credibility trees based on subtype C sequences of HIV-1 *p17* fragment (**A**) and subtype 3b sequences of HCV *NS5B* fragment (**B**) among HIV-1/HCV coinfected drug users in Yingjiang, Kaiyuan Prefectures, Yunnan Province, China. The different colored lines indicate the sequences from different geographical locations, and the black spots indicate the nodes of major lineages. The ages of nodes, with 95% confidence interval, and posterior probability are indicated beside the major nodes

A maximum clade credibility tree based on HIV-1 subtype C *p17* fragments showed that HIV-1 among drug users in Kaiyuan was introduced from Yingjiang in 2004 and then spread to other prefectures of Yunnan Province (Fig. 4). The results based on HCV 3b *NS5B* fragments showed that HCV among drug users in Kaiyuan was introduced from Yingjiang through multiple lineages in 2007 and then spread to other prefectures of Yunnan Province (Fig. 4). These results indicated that the transmission of HIV-1 and HCV among HIV-1/HCV coinfected patients had high consistency.

## Discussion

Due to high-risk behaviors such as drug injection and sharing of needles, drug users, especially those who inject drugs, are at high risk of HIV-1 transmission in China. The first cases of HIV-1 in China were found in drug users of Yunnan province [16], and then HIV-1 spread in China rapidly. By the end of 2017, the cumulative number of HIV-1-positive individuals reported in China was 718,270. Among these HIV-1-infected cases, a high proportion are transmitted through drug users. Data from the 1995 to 2011 China National AIDS Case Report and Sentinel Surveillance System showed that the rate of HIV-1 infection among drug users increased rapidly before 2000. After 2004, the rate of HIV-1 infection among drug addicts declined, with an infection rate of 6.4% in 2011 [17]. In China, there are differences in HIV-1 infection rates in different regions. In areas close to the “Golden Triangle” and along drug trafficking routes, such as Xinjiang and Yunnan, HIV-1 infection among drug users will reach 40% [18, 19]. Similar to HIV-1, HCV can also transmit among drug users and is considered to be the most common viral infection among drug users [20]. Systematic analysis showed that the prevalence of HCV in Chinese drug addicts reached 50% [21, 22], while the HCV infection rate in Yunnan was 90% [23]. As HIV-1 and HCV share similar transmission routes, HIV-1/HCV coinfection is more common among drug users, while HIV-1/HCV coinfection makes the management of infection in drug users more complicated. The coinfection rate of HIV-1/HCV is not consistent in different regions. The data showed that the highest rate of coinfection was 46.3% in Yunnan, followed by 36.2% in Xinjiang [18].

Yunnan province is located in southwestern China and situated along drug trafficking routes that channel heroin into China. Laos, Vietnam and Thailand all border this province. Dehong prefecture is bordered by Myanmar, and Honghe prefecture borders Vietnam. In this study, the prevalence of HIV-1/HCV coinfection was 32% and 56% among drug users of Yingjiang and Kaiyuan prefectures, Yunnan province, respectively.

The main subtypes of HIV-1 circulating in drug users in 1990 were B, C and CRF01_AE [24, 25]. From 2000 to 2001, a study analyzed HIV-1 subtypes of PWIDs of Dehong, Honghe and Wenshan prefectures, Yunnan province. The results showed that the main HIV-1 subtype in Dehong was URFs (71%), and the other subtype was B, while in Honghe and Wenshan, CRF 08_BC was the most prevalent [26]. At the same time, a new recombinant comprising CRF07_BC and CRF08_BC was also found in Honghe prefecture [27]. A study also showed that there are more URFs among drug users [28]. In this study, we found a high prevalence of HIV-1 CRF08_BC and URF_BC recombinants among HIV-1/HCV coinfected drug users in Kaiyuan province and Yingjiang prefecture, Yunnan province, respectively, which was similar to previous studies. HCV genotypes 3 and 6 were common among drug users in Yunnan province, and a higher rate of HCV genotype 6 was seen in recent years [23, 29, 30]. We also concluded that HCV 3b was the most predominant among HIV-1/HCV coinfected drug users in Yingjiang and Kaiyuan prefectures, Yunnan province. In addition, a high proportion of HCV genotype 6 was found in this population.

A previous study discovered that HCV prevalence may predict the HIV-1 epidemic [31], and another study illustrated that individuals with HIV-1 virus subtypes that clustered with those of HIV-1/HCV coinfected patients had a higher risk for acquiring HCV [32]. These studies all indicate that HIV-1 and HCV may have some correlation. Our study further investigated the spatial dynamics of HIV-1 and HCV by performing phylogeographic analyses. The results showed that the transmission of the two viruses was the same. This finding can help us predict the transmission route of one virus based on the known route of another virus to some extent.

## Conclusions

In summary, the present study explored the subtypes and spatial dynamics of HIV-1 and HCV among HIV-1/HCV coinfected drug users from Yingjiang and Kaiyuan prefectures, Yunnan province. Phylogenetic analysis based on partial HCV *C/E2*, *NS5B* and HIV-1 *p17*, *pol* and *env* genes revealed that HCV 3b and HIV-1 CRF08_BC and URF_BCs were predominant in this risk group. Furthermore, phylogeographic analysis confirmed that the transmission patterns of HIV-1 and HCV were highly consistent, providing new insights into the prevention of HIV-1 and HCV.

## Supplementary Information

**Additional file 1: Table S1.** The reference sequences of HCV *NS5B* fragments that downloaded from GenBank and used for Bayesian phylogeographic analysis.

**Additional file 2: Table S2.** The reference sequences of HIV-1 *p17* fragments that downloaded from GenBank and used for Bayesian phylogeographic analysis.

**Additional file 3: Table S3.** The amplification results of HIV-1 and HCV fragments among HIV-1/HCV coinfected drug users in Yingjiang and Kaiyuan prefectures, Yunnan, China.

**Additional file 4: Figure S1.** The maximum-likelihood trees based on *env* and *p17* fragments of HIV-1 among HIV-1/HCV coinfected drug users in Yunnan Province, China. The red triangles and the blue circles indicate the sequences that amplified from drug users in Yingjiang and Kaiyuan Prefectures, respectively. The different colored sectors indicate the sequences with different subtypes.

## Data Availability

The datasets generated during the current study are available in the GenBank, https://www.ncbi.nlm.nih.gov/, with accession numbers MF990907-MF991024. MG334013-MG334120, MG385895-MG386024, MG432013-MG432106, MG450396-MG450541, MG461702-MG461853, MG494384-MG494479, MG549076-MG549188, MG549193-MG549305, MG763455- MG763560. All the original data are available from the corresponding author as required.
