# Supplementary material for: Consistency of spatial dynamics of HIV-1 and HCV among HIV-1/HCV coinfected drug users in China
Source: BMC Infect Dis. 2021 Sep 25;21:1001. doi: 10.1186/s12879-021-06711-6 (PMC8465760; doi:10.1186/s12879-021-06711-6)
Supplement: Supplementary file 1 — Additional file 1: Table S1. The reference sequences of HCV NS5B fragments that downloaded from GenBank and used for Bayesian phylogeographic analysis. [file 12879_2021_6711_MOESM1_ESM.docx]

Table S1. The reference sequences of HCV *NS5B* fragments that downloaded from GenBank and used for Bayesian phylogeographic analysis.

| GenBank accession number | Sample site |
| --- | --- |
| KT735720 | Dali, China |
| KT735724 | Dali, China |
| KT735728 | Dali, China |
| KT735730 | Dali, China |
| KT735735 | Dali, China |
| KT735737 | Dali, China |
| KT735740 | Dali, China |
| KT735741 | Dali, China |
| KT735746 | Dali, China |
| KT735791 | Kunming, China |
| KT735796 | Kunming, China |
| KT735798 | Kunming, China |
| KT735806 | Kunming, China |
| KT735807 | Kunming, China |
| KT735808 | Kunming, China |
| KT735817 | Kunming, China |
| KT735818 | Kunming, China |
| KT735825 | Kunming, China |
| KT735826 | Lincang, China |
| KT735828 | Lincang, China |
| KT735831 | Lincang, China |
| KT735832 | Lincang, China |
| KT735836 | Lincang, China |
| KT735837 | Lincang, China |
| KT735840 | Lincang, China |
| KT735843 | Lincang, China |
| KT735847 | Lincang, China |
| KT735848 | Lincang, China |
| KT735850 | Lincang, China |
| KT735851 | Lincang, China |
| KT735852 | Lincang, China |
| KT735854 | Lincang, China |
| KT735856 | Lincang, China |
| KT735858 | Lincang, China |
| KT735859 | Lincang, China |
| KT735864 | Wenshan, China |
| KT735865 | Wenshan, China |
| KT735869 | Wenshan, China |
| KT735875 | Wenshan, China |
| KT735879 | Wenshan, China |
| KT735882 | Wenshan, China |
| KT735886 | Wenshan, China |
| KT735889 | Wenshan, China |
| KT735894 | Wenshan, China |
| KT735895 | Wenshan, China |
| KT735897 | Wenshan, China |
