# Supplementary material for: Consistency of spatial dynamics of HIV-1 and HCV among HIV-1/HCV coinfected drug users in China
Source: BMC Infect Dis. 2021 Sep 25;21:1001. doi: 10.1186/s12879-021-06711-6 (PMC8465760; doi:10.1186/s12879-021-06711-6)
Supplement: Supplementary file 2 — Additional file 2: Table S2. The reference sequences of HIV-1 p17 fragments that downloaded from GenBank and used for Bayesian phylogeographic analysis. [file 12879_2021_6711_MOESM2_ESM.docx]

Table S2. The reference sequences of HIV-1 *p17* fragments that downloaded from GenBank and used for Bayesian phylogeographic analysis.

| GenBank accession number | Sample site |
| --- | --- |
| AF067155 | India |
| AY772699 | South Africa |
| JX263542 | Kunming, China |
| JX263546 | Kunming, China |
| JX263547 | Kunming, China |
| JX263566 | Wenshan, China |
| JX263568 | Wenshan, China |
| JX263569 | Wenshan, China |
| JX263570 | Wenshan, China |
| JX263574 | Wenshan, China |
| JX263575 | Wenshan, China |
| JX263576 | Chuxiong, China |
| JX263577 | Chuxiong, China |
| JX263578 | Chuxiong, China |
| JX263583 | Wenshan, China |
| JX263586 | Kunming, China |
| JX263591 | Yuxi, China |
| JX263595 | Qujing, China |
| JX263598 | Dali, China |
| JX263603 | Lijiang, China |
| JX263606 | Lincang, China |
| JX263608 | Lincang, China |
| JX263610 | Lincang, China |
| JX263611 | Lincang, China |
| JX263615 | Qujing, China |
| JX263618 | Banna, China |
| JX263619 | Banna, China |
| JX263620 | Yuxi, China |
| JX263621 | Nujiang, China |
| JX263622 | Zhaotong, China |
| JX263625 | Zhaotong, China |
| JX263631 | Baoshan, China |
| JX263640 | Kunming, China |
| MF503170 | Zhaotong, China |
| MF503171 | Zhaotong, China |
| MF503172 | Zhaotong, China |
| MF503174 | Zhaotong, China |
| MF503175 | Zhaotong, China |
| MF503176 | Zhaotong, China |
| MF503177 | Zhaotong, China |
| MF503178 | Zhaotong, China |
| MF503179 | Zhaotong, China |
| MF503181 | Zhaotong, China |
| MF503182 | Zhaotong, China |
| MF503183 | Zhaotong, China |
| MF503184 | Zhaotong, China |
| MF503185 | Zhaotong, China |
| MF503186 | Zhaotong, China |
| MF503187 | Zhaotong, China |
| MF503188 | Zhaotong, China |
| MF503190 | Zhaotong, China |
| MF503191 | Zhaotong, China |
| MF503198 | Zhaotong, China |
| MF503203 | Zhaotong, China |
| MF503210 | Zhaotong, China |
| MF503212 | Zhaotong, China |
| MF503213 | Zhaotong, China |
| MF503216 | Zhaotong, China |
| MF503218 | Zhaotong, China |
| MF503219 | Zhaotong, China |
| MF503222 | Zhaotong, China |
| MF503227 | Zhaotong, China |
| MF503228 | Zhaotong, China |
| MF503229 | Zhaotong, China |
| MF503230 | Zhaotong, China |
| MF503233 | Zhaotong, China |
| MF503235 | Zhaotong, China |
| MF503236 | Zhaotong, China |
| MF503237 | Zhaotong, China |
| MF503238 | Zhaotong, China |
| MF503241 | Zhaotong, China |
| U46016 | Ethiopia |
| U52953 | Brazil |
