# Supplementary material for: Consistency of spatial dynamics of HIV-1 and HCV among HIV-1/HCV coinfected drug users in China
Source: BMC Infect Dis. 2021 Sep 25;21:1001. doi: 10.1186/s12879-021-06711-6 (PMC8465760; doi:10.1186/s12879-021-06711-6)
Supplement: Supplementary file 3 — Additional file 3: Table S3. The amplification results of HIV-1 and HCV fragments among HIV-1/HCV coinfected drug users in Yingjiang and Kaiyuan prefectures, Yunnan, China. [file 12879_2021_6711_MOESM3_ESM.docx]

Table S3. The amplification results of HIV-1 and HCV fragments among HIV-1/HCV coinfected drug users in Yingjiang and Kaiyuan prefectures, Yunnan, China.

| ART^*^ status of HIV-1 |  | Jingjiang |  |  | Kaiyuan |  |
| --- | --- | --- | --- | --- | --- | --- |
|  | Total participants | HIV-1 fragments | HCV  fragments | Total participants | HIV-1 fragments | HCV  fragments |
| ART | 26 | 17(65.4) | 25(96.2) | 42 | 27(64.3) | 35(83.3) |
| ART naïve | 33 | 33(100.0) | 31(93.9) | 62 | 55(88.7) | 53(85.5) |
| Unknown^#^ | 68 | 64(94.1) | 59(92.2) | 79 | 46(58.2) | 66(83.5) |

^*^ Antiretroviral therapy; ^#^ People who had not answered the questions regarding the status of ART of HIV-1.
