# Supplementary figures and images for: Consistency of spatial dynamics of HIV-1 and HCV among HIV-1/HCV coinfected drug users in China
Source: BMC Infect Dis. 2021 Sep 25;21:1001. doi: 10.1186/s12879-021-06711-6 (PMC8465760; doi:10.1186/s12879-021-06711-6)

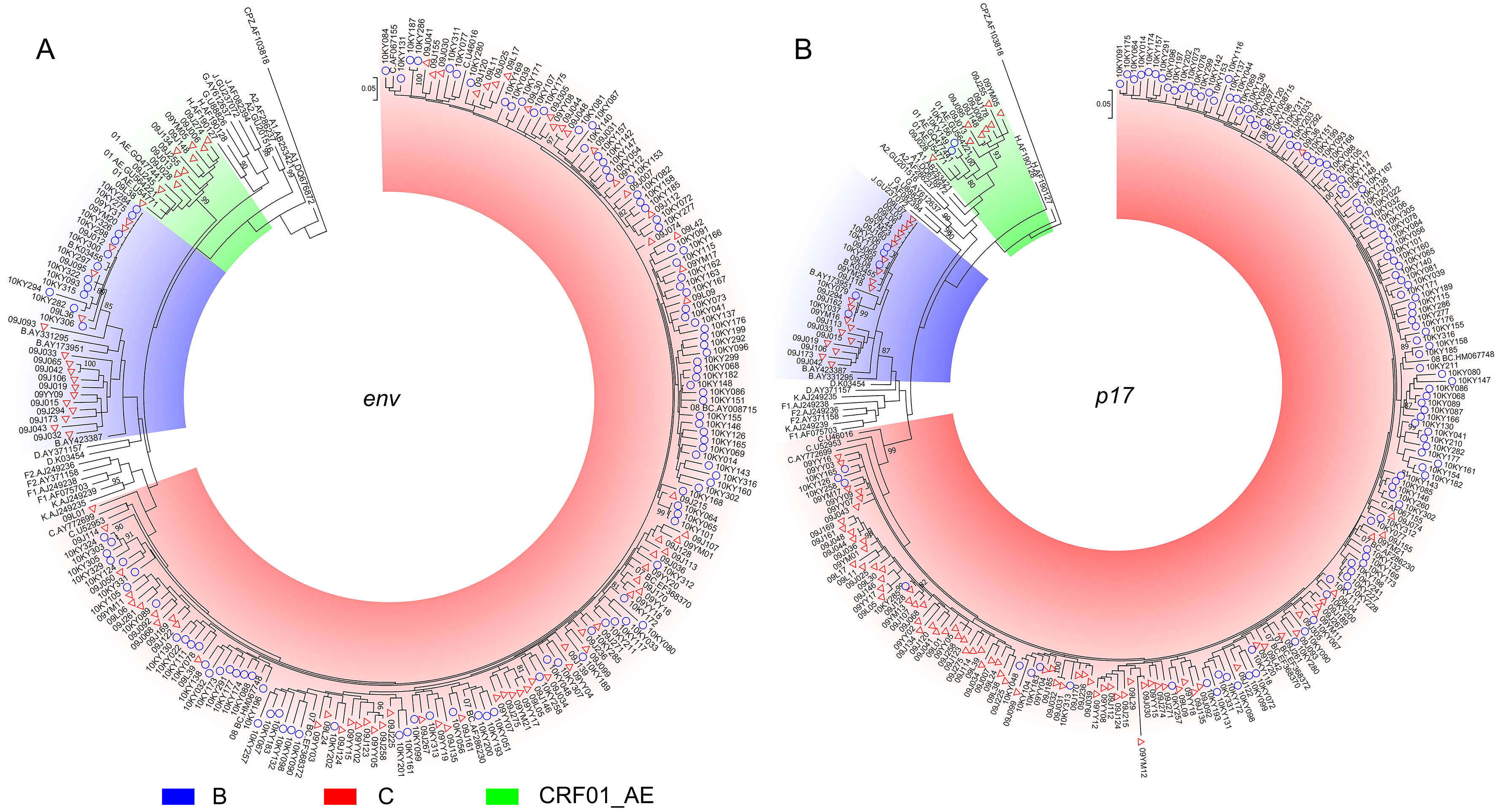

Supplement: Supplementary file 4 — Additional file 4: Figure S1. The maximum-likelihood trees based on env and p17 fragments of HIV-1 among HIV-1/HCV coinfected drug users in Yunnan Province, China. The red triangles and the blue circles indicate the sequences that amplified from drug users in Yingjiang and Kaiyuan Prefectures, respectively. The different colored sectors indicate the sequences with different subtypes. [file 12879_2021_6711_MOESM4_ESM.tif]
